# Neuronal desertification after a direct lightning strike: a case report

**DOI:** 10.1186/s13256-022-03500-w

**Published:** 2022-10-21

**Authors:** Erik Roman-Pognuz, Edoardo Moro, Elisabetta Macchini, Edoardo Di Paolo, Kenneth Pesenti, Umberto Lucangelo, Rossana Bussani, Elisa Baratella, Tommaso Pellis, Giuseppe Ristagno

**Affiliations:** 1 Department of Surgical Medical and Health Sciences, University of Trieste, Trieste, Italy; 2 Department of Anesthesia and Intensive Care, Integrated University Health Authority of Trieste, Trieste, Italy; 3 Department of Pathology, Integrated University Health Authority of Trieste, Trieste, Italy; 4 Department of Radiology, Integrated University Health Authority of Trieste, Trieste, Italy; 5 Health care company n.5 “Western Friuli” (ASS 5), Santa Maria degli Angeli Hospital in Pordenone, Pordenone, Italy; 6 IRCCS Foundation Maggiore Policlinico Hospital, Milan, Italy

**Keywords:** Cardiac arrest, Lightning strike, Neuronal desertification, Brain injury, Hypoxic ischaemic brain injury

## Abstract

**Background:**

Lightning strike is a rare but dramatic cause of injury. Patients admitted to intensive care units (ICUs) with lightning strike frequently have a high mortality and significant long-term morbidity related to a direct brain injury or induced cardiac arrest (CA).

**Case presentation:**

A 50-year-old Caucasian man was admitted to our hospital after being struck by lightning resulting in immediate CA. Spontaneous circulation was initially restored, and the man was admitted to the ICU, but ultimately died while in hospital due to neurological injury. The computer tomography scan revealed a massive loss of grey-white matter differentiation at the fronto-temporal lobes bilaterally. Somatosensory-evoked potentials demonstrated bilateral absence of the cortical somatosensory N20-potential, and the electroencephalogram recorded minimal cerebral electrical activity. The patient died on day 10 and a post-mortem study revealed a widespread loss of neurons.

**Conclusion:**

This case study illustrates severe brain injury caused by a direct lighting strike, with the patient presenting an extraordinary microscopic pattern of neuronal desertification.

## Background

Lightning occurs nearly 50 times per second worldwide [1], and although injuries secondary to lightning strike are rare, they are associated to significant long-term morbidity and high mortality. Many lightning strikes result in immediate death due to fatal arrhythmias or respiratory failure due to critical alterations in the cardiovascular, respiratory, and central nervous systems [2]. However, the success of resuscitation attempts may be higher in people struck by lightning compared with other etiologies of cardiac arrest (CA), leading to a return to spontaneous circulation (ROSC) and, in rare cases, good functional outcome. We report here the case of a middle-aged man struck by lightning and review the literature on the pathophysiology of lightning-induced injury. In addition, we describe a pattern of diffuse cerebral neuronal desertification revealed at the post-mortem study that was directly related to the injury.

## Case presentation

**Fig. 1:**
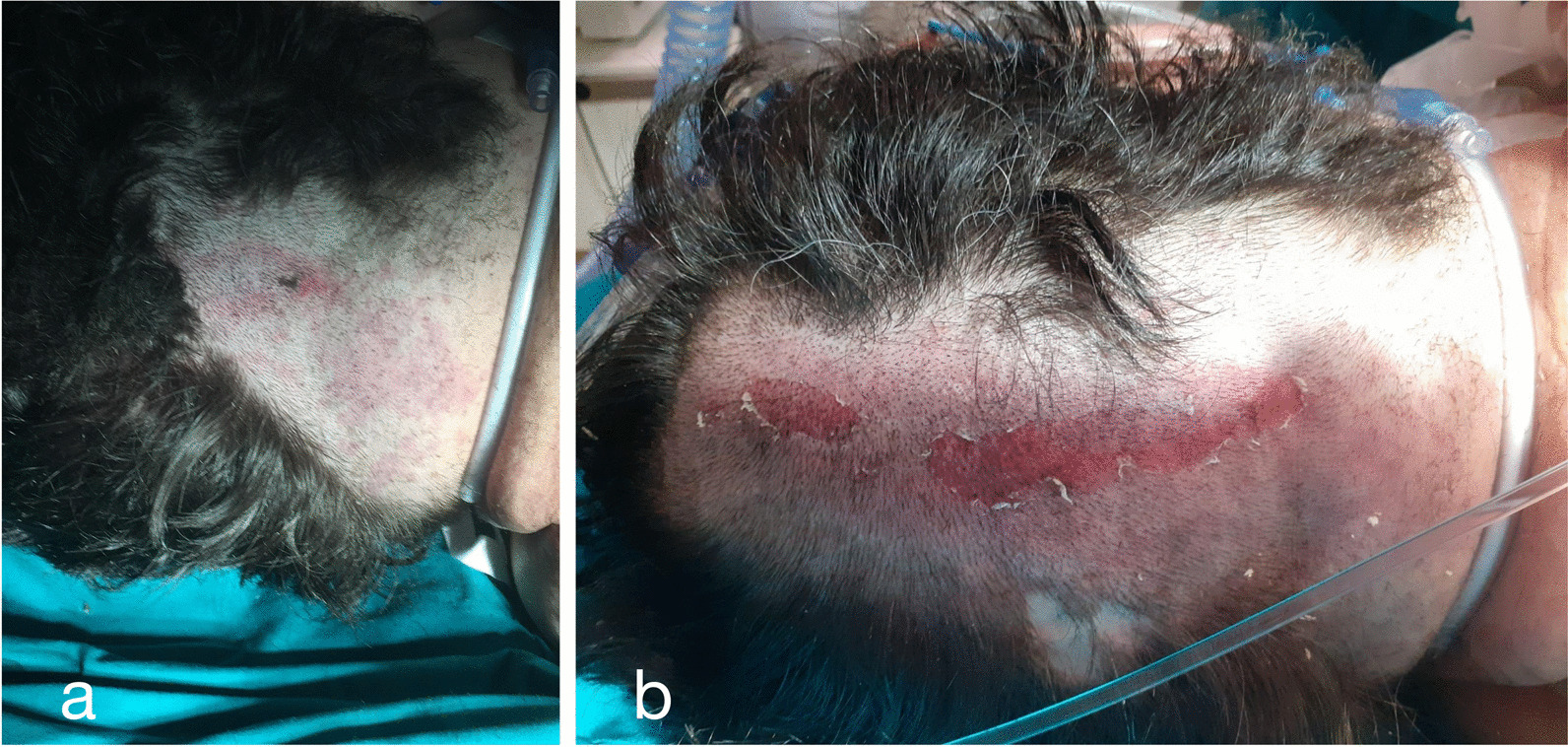
**a**, **b** Round black eschar, presumably where the lightning entered the body. **b** Eschar from a different view

During a summer rainstorm, a 50-year-old Caucasian man riding a bicycle in the forested area around the city of Trieste, Italy, was struck by lightning, resulting in immediate CA, as reported by bystanders. Basic life support was not initiated by the lay bystanders, but was initiated only a few minutes later by a physician hiking nearby and subsequently continued by the medical emergency system personnel who arrived at the scene 15 min after the event. The presenting rhythm was asystole, with a downtime before return of spontaneous circulation (ROSC) of 16 minutes. After ROSC, the patient remained unconscious with a Glasgow Coma Score (GCS) of 3, non-reactive mydriatic pupils, and stable blood pressure and oxygenation values once intubated. During the secondary examination at hospital admission, a cutaneous lesion in the occipital area was noted, described as singed hair with a dominant burning smell, linear in shape, with a small round black eschar, presumably compatible with the first contact with lightning (Fig. 1).

**Fig. 2:**
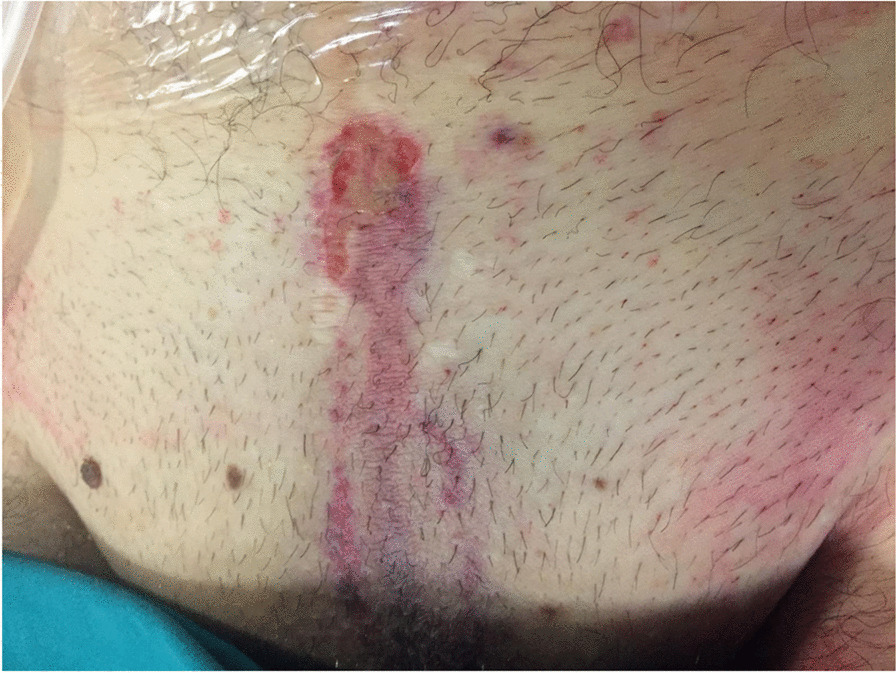
Erythematous cutaneous lesions with singed hair at the suprapubic level likely due to the output area

The presence of burnt plastic at the level of the occipital slot of the helmet was consistent with the hypothesis of the eschar being the first contact with lightning. Similar erythematous cutaneous lesions with singed hair were found at the suprapubic level, probably the output area in proximity to the metal core of the bicycle frame through the saddle (Fig. 2).

**Fig. 3:**
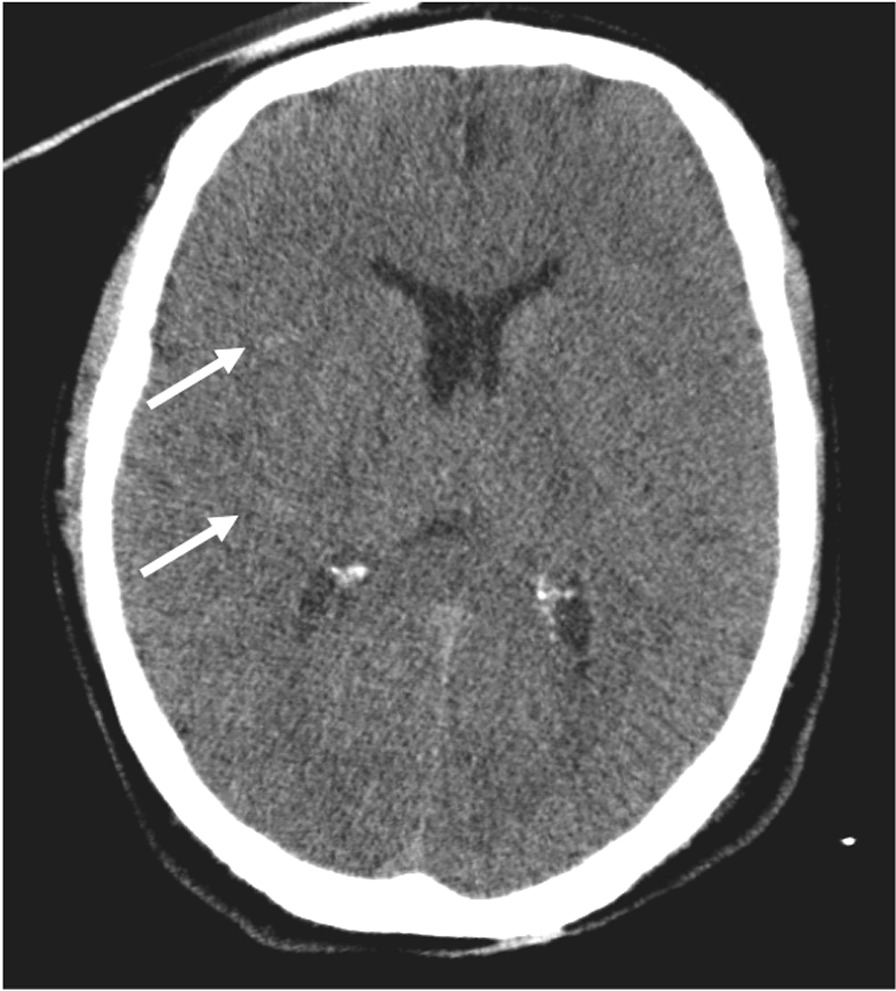
Axial non-enhanced brain computed tomography scan demonstrates a loss of grey-white matter differentiation at the fronto-temporal lobes bilaterally with gyral effacement. Subtle subarachnoid hemorrhage (white arrows) is also noted

In addition, the right tympanic membrane was perforated. Ophthalmological assessment was negative. Total body computed tomography (CT) scan showed a modest right apical and temporal subarachnoid hemorrhage, millimeter-sized petechial hemorrhages in the cortical-subcortical junction of the fronto-parietal lobes, and slight signs of initial edema (Fig. 3). An abundant hemoperitoneum associated with a bleeding hepatic angioma, was present.

**Fig. 4:**
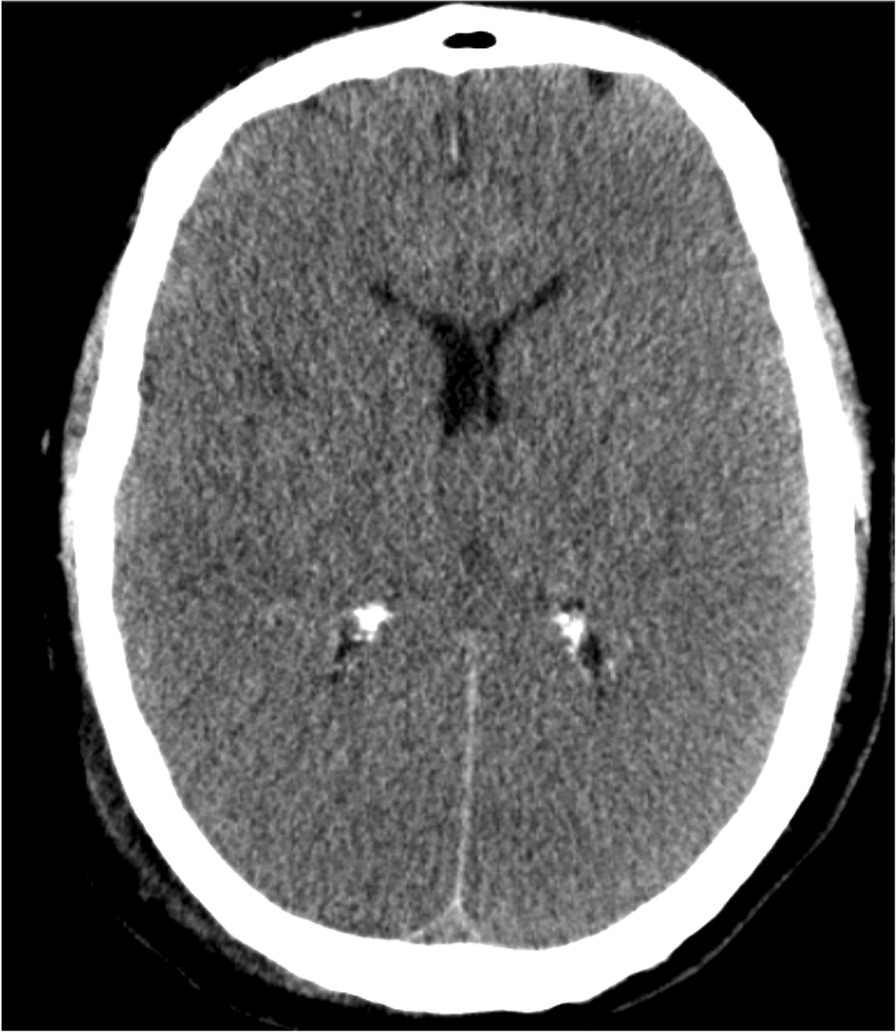
Computed tomography scan performed 4 days after the first scan shows diffuse cortical hypodensity of both cerebral hemispheres, with a more evident loss of grey-white matter differentiation with gyral effacement and compression of lateral ventricles

The patient underwent immediate damage control surgery, with abdominal packaging to control the bleeding, and then was admitted to our intensive care unit (ICU). Laboratory tests on blood samples revealed elevated biomarkers of myocardial and hepatic injury (high-sensitivity troponin I, 90,879 ng/L; creatine kinase, 1131 U/L; aspartate and alanine transaminases, 361 and 421 U/L, respectively; blood lactate, 11.36 mmol/L). The electrocardiogram (EKG) showed sinus tachycardia with minimal nonspecific ST elevation in the inferior leads, and cardiac ultrasonography showed normokinetic ventricles; no further cardiology investigations were undertaken at that time. Owing to the prolonged time to admission, target controlled temperature management was not established, and the subsequent appearance of fever (from day 2 after ICU admission, mean central body temperature of 38.2 °C with only one peak at 38.8 °C) was managed with ice packs, a short period of diclofenac infusion, and then acetaminophen on demand. No prophylactic therapy against vasospasm or brain edema was initiated since serum hyperosmolality was persistently elevated (319 mOsm/L). Following surgery, the patient remained in cardiogenic shock with profound hemodynamic instability and lactic metabolic acidosis. Increasingly higher norepinephrine doses were needed to support arterial pressure, peaking 0.95 mcg/kg/min on day 2. On day 3, after adequate goal-direct volume expansion, terlipressin infusion was added to the treatment regimen (peaking at 1.28 mcg/kg/h). Cardiac function was restored to normal, as demonstrated by a further ultrasound assessment. Acute kidney injury due to rhabdomyolysis (noted at admission to the ICU by the elevated levels of serum creatinine, blood urea nitrogen, and serum creatine kinase [1131 U]) and oliguria were managed with volume expansion with crystalloids, urine alkalinization with sodium bicarbonate boluses, and diuretics. A brain CT scan was repeated on day 2, revealing a further reduction of the cortico-subcortical differentiation bilaterally in the frontal-parietal and temporo-occipital areas compatible with anoxic edema, a marked flattening of the cortical convolutions, and the obliteration of the supratentorial peri-encephalic liquor spaces, also at the level of basal cisterns. On day 3, sedation was interrupted to allow for neuroprognostication. A neurological clinical assessment revealed a persisting GCS of 3 and the absence of pupillary and corneal reflexes. Somatosensory evoked potentials demonstrated bilateral absence of the cortical somatosensory N20-potential (ERB- and N13-potential were present) and the electroencephalogram (EEG) recorded minimal cerebral electrical activity with very low voltages. On day 4, hemodynamic stability was obtained, allowing for tapering of vasopressor support, and the fever ceased. On day 5, the patient remained unconscious with a GCS of 3 and only a weak cough reflex and respiratory drive. A new EEG revealed an isoelectric trace. A cerebral CT angiography revealed a regular opacification of the arterial circulation with normal venous drainage. Direct scanning highlighted further reduced cortical-subcortical differentiation and marked flattening of brain convolution (Fig. 4).

**Fig. 5:**
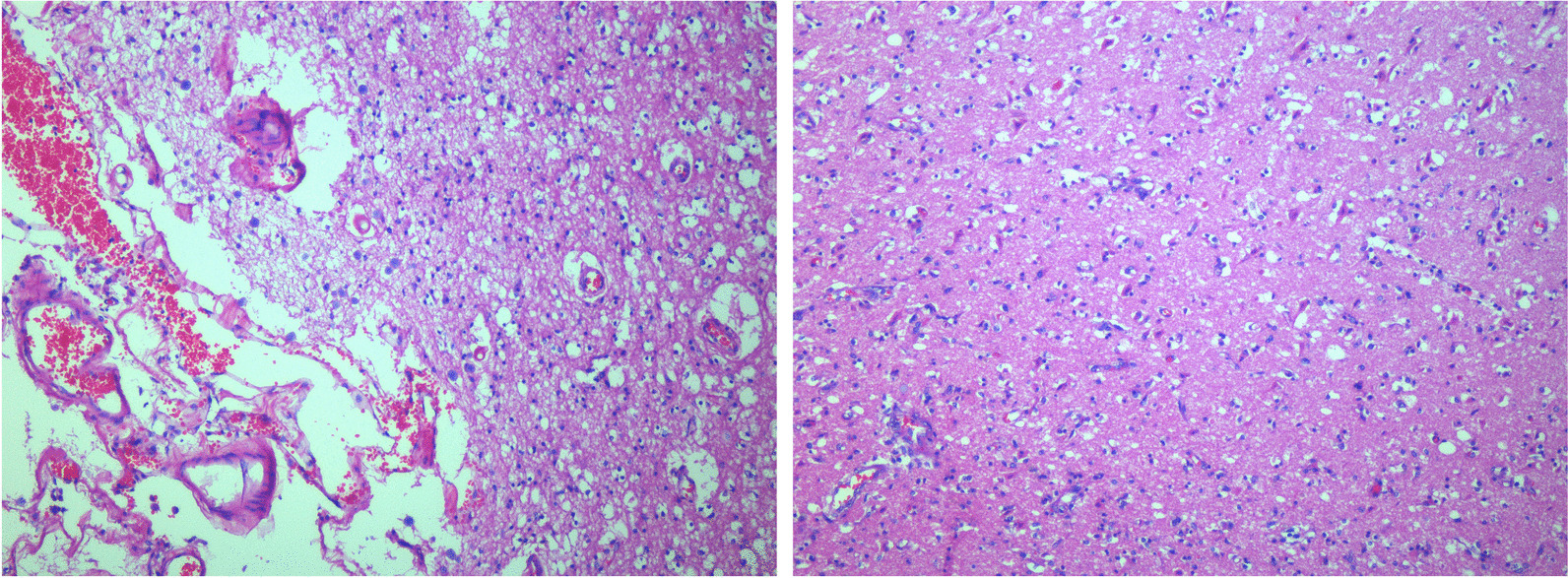
Cerebral parenchymal sections with an abundant, non-selective loss of neuronal elements

The patient died on day 10. A post-mortem evaluation revealed signs of multi-organ hypoperfusion, with effects on subendocardial and transmural cardiomyocytes. Cerebral sections confirmed the CT findings of petechiae and subarachnoid hemorrhage previously located along the electrical current and as a result of direct damage to brain vasculature. Brain tissue softening and edema were associated with the direct effects of electrical passage. The cerebral parenchyma sections revealed an abundant, non-selective loss of neurons (Fig. 5). We collected detailed medical information from the patient’s relatives, but nothing in his medical history could be related to any previous neurological or cognitive disturbances.

## Discussion and conclusion

Lightning strike is responsible for an average of 300 injuries and 100 deaths per year in the USA [3–5], although this figure is likely to be an underestimate, with lightning-related fatalities and hospitalizations most likely to be more common than reported in the literature [6]. Approximately 30% of those struck by lightning die, and up to 74% of survivors may have permanent disabilities [4, 7, 8]. Two-thirds of lightning-associated deaths occur within 1 hour of the injury and are generally due to a fatal arrhythmia or respiratory failure [9, 10] as a result of asystole or ventricular fibrillation [11, 12]. Direct strikes are more likely to result in asystole due to sudden depolarization of the heart [2, 11]. Ventricular fibrillation or tachycardia may also be frequently associated with lightning [13], but they are more likely the consequence of indirect strikes [11]. This case report describes the dramatic neurological consequences of a direct lightning strike in a patient who survived the initial strike. Of particular note, we present the pathology findings determined over time by the effects of electrical current passing through the brain, namely, massive neuronal loss leading to cerebral neuronal desertification. Pathologists reported a non-selective depletion of neuronal cells in the brain parenchymal sections studied.

The alterations described in our patient are common findings in patients with Alzheimer’s disease, which is a neurodegenerative disease that occurs over years due to the progressive formation, diffusion and thickening of diffuse and senile plaques. Spongiosis in neurons results in the death of the affected neurons, visible as an empty halo on brain tissue sections. Gliosis is the reactive proliferation of the common glia in situations of deneuronization. Perivascular hali are observed, resulting from receding brain tissue and the neuropil, as well as from gas bubbles due to electrolytic processes. What we observed in our patient usually happens over months in those with severe cerebral ischemia and over years in those with dementia. The neurodegeneneration observed in our patient was condensed into a few days and can be explained only by an external event that exerted a dramatic influence on cell tropism and, consequently, cell death. The combination of both CA and direct electrical damage might have resulted in an extremely rapid and complete cerebral crushing in this specific case.

Our patient suffered damage due to the global cerebral ischemia that developed during the no-flow and low-flow phases after the CA, but he also suffered significant injuries that may have been due to the direct lightning strike. A sudden and extremely high warming-up of the brain due to the route of the lightning through the head indubitably contributed to immediate neuronal death and subsequent increased injury in the re-perfused “warm” brain. Indeed, neurons transmit physiologically to each other through chemical and electrical synapses. The strength of the electrical discharge of the lightning strike may have immediately altered both processes, in particular the electrical inter-neuronal communication pathway [14].

 Lightning strikes cause injuries through different mechanisms [1–15]. A direct strike is a lightning bolt that directly hits a person, and it this was the main mechanism of injury in the patient reported here. Survival of persons after a direct lightning strike who suffer CA is a rare event. The severity of an electric injury is directly correlated to the intensity of the current that crosses through the person's tissue [16], which is dependent on the electrical resistance of the different tissues. Bone offers the highest resistance, while muscles, vessels, and nerves provide the lowest. The skin is the main barrier against penetration of the electrical current in the body, but its resistance is significantly impaired by the presence of moisture, such as sweat or water, on its surface [2]. 

Injuries due to electricity occur by four mechanisms: (1) light; (2) direct effect of electrical current on body tissues; (3) conversion of electrical energy to thermal energy, resulting in deep and superficial burns; and (4) blunt mechanical injury from lightning strike, muscle contraction, or as a complication of a fall after electrocution [17]. The light component of electricity can injury the eye, causing delayed damage such as cataracts (the most common finding) but also more serious and acute conditions such as macular holes or edema, optic nerve neuropathy, and retinal detachment. In contrast, the temperature component, with temperatures that can be as high as 8000 °C for a few milliseconds, is responsible for skin and hair burns. The electrical component accounts for damage to the nervous system and heart, and for the Lichtenberg figures and tip-toe signs. Neuron and cardiomyocyte death is believed to occur through the cellular mechanism of electroporation: light can cause the reorganization of the lipid layer with the generation of pores due to modification of the membrane potential. As a result, cells die from the progressive depletion of energy spent to maintain the ion gradient [8]. In the case of a direct strike, tissues are severely damaged by the consistent amount of electric charge absorbed by the individual who has been struck [18, 19]. Similar to the presentation of our case, people hit by lightning may present with pupils that are fixed and dilated or asymmetric due to autonomic dysfunction. Consequently, fixed, dilated, or asymmetric pupils should not be used as the rationale to stop resuscitation [9]. Motor system diseases and movement disorders are part of this delayed syndrome. The last pattern is secondary to other mechanisms and includes subdural and epidural hematomas and subarachnoid hemorrhage originating from trauma [8–21]. Brain lesions are the most common finding at autopsy, whereas cerebellar injuries and injury to peripheral nerves are rare [21]. Different types of hemorrhages and ischemic encephalopathy are widely described [22]. Huan-Jui *et al*. [23] reported a right frontotemporal infarction also involving the basal ganglia in a person who suffered a low alternating voltage shock, and Eriksson *et al*. [24] reported pons infarction in a person injured by lightning; nevertheless, cerebral infarction is a rare finding, and is commonly linked to vascular damage. Gliomas and cerebral atrophy could also be permanent consequences of lightning shock [21]. Only a small number of cerebellar syndromes are reported in the literature [21], all with a notable form of acute atrophy [25] secondary to extensive brain damage; abrupt cerebral salt wasting syndrome has also been reported [8–17].

 The clinical manifestations of electrical injuries range from mild superficial skin burns to severe multiorgan dysfunction and death. The case we present is an example of severe multiorgan involvement, with the patient presenting with CA with first monitored rhythm asystole, either due to massive myocardial depolarization or as a degradation of ventricular fibrillation due to several minutes of no-flow downtime. Fixed dilated pupils, as exhibited in our case, are also a possible manifestation of electrical injury. The underlying mechanism has been attributed to autonomic dysfunction and may represent an important confounder of prognostication following central nervous injury or cardiac arrest, and should not be used as a reason to stop resuscitation [26]. A thorough examination of the patient showed tympanic perforation, which should be sought as it has been reported in 50–80% of patients struck by lightning [27]. Ruptured tympanic membranes are secondary to barotrauma determined by the peak temperature within a bolt of lightning, which rises within milliseconds to 30,000 K, generating a shock wave of up to 20 atmospheres induced by the rapid heating of the surrounding air [9]. This shock wave then can be transmitted through the body and result in mechanical trauma. Thus, damage to internal organs, albeit uncommon, is possible as reported in this case and should be excluded.

 We also observed rhabdomyolysis that may result from massive tissue necrosis and which was complicated by pigment-induced acute kidney injury. We observed superficial thermal burns that were limited in extension and that went unnoticed at the primary survey. Burns are most common at the site of electrical contact and at parts of the body in contact with the ground at the time of injury. The degree of external injury cannot be used to determine the extent of internal damage. Indeed, seemingly minor surface burns may coexist with massive muscle coagulation and necrosis, as well as internal organ injury. Neurological injury was the cause of death of our patient. Vascular and parenchymal injuries of the central nervous system are often associated with peripheral and autonomic nervous system dysfunction. In our case report we describe the consequence of a direct lightning strike on the neural cells. Cerebral multicentric necrosis, particularly of the basal ganglia, was associated with massive cerebral edema and large areas of absolute neuronal absence, described as desertification. The latter describes a major reduction in the number of neuronal elements, not selective by type of neuron, that closely resembles the rarefaction commonly seen in persons with chronic vascular dementia [28]. These conditions would be secondary to the dramatic acute insult that the patient suffered both directly by the lightning strike and secondary by edema due to the prolonged CA. To date, acute atrophy has been reported in only a small number of cerebellar syndromes secondary to lightning shock [24].

## Conclusion

We present a fatal case caused by a direct lightning strike, with the patient presenting with CA followed by initial resuscitation and death due to severe direct neurological injury, with an impressive microscopic pattern of neuronal desertification. Possible mechanisms of neuronal injury in this case include the direct high warm-up of the brain caused by the lightning and a subsequent increased injury due to the re-perfused “warm” brain after the CA.

## Data Availability

All data generated or analyzed during this study are included in this published article

## Abbreviations

CA: Cardiac arrest
CT: Computer tomography
EEG: Electroencephalogram
EKG: Electrocardiogram
GCS: Glasgow Coma Scale
ICU: Intensive care unit
ROSC: Return on sustained circulation

## References

[1] Davis C, Engeln A, Johnson EL, *et al*. Wilderness Medical Society practice guidelines for the prevention and treatment of lightning injuries: 2014 update. Wilderness Environ Med. 2014;25:S86-95. 10.1016/j.wem.2014.08.011 25498265

[2] Koumbourlis A. Electrical injuries. Crit Care Med. 2002;30:424–30. 10.1097/00003246-200211001-00007 12528784

[3] Spies C, Trohman RG. Narrative review: electrocution and life-threatening electrical injuries. Ann Intern Med. 2006;145:531–7. 10.7326/0003-4819-145-7-200610030-00011 17015871

[4] Zafren K, Durrer B, Herry JP, Brugger H. Lightning injuries: prevention and on-site treatment in mountains and remote areas. Official guidelines of the International Commission for Mountain Emergency Medicine and the Medical Commission of the International Mountaineering and Climbing Federation (ICAR and UIAA MEDCOM). Resuscitation. 2005;65:369–72. 10.1016/j.resuscitation.2004.12.014 15919576

[5] Centers for Disease Control and Prevention (CDC). Lightning-associated injuries and deaths among military personnel—United States, 1998–2001. MMWR Morb Mortal Wkly Rep. 2002;51:859–62. 12363338

[6] López RE, Holle RL, Heitkamp TA, Boyson M, Cherington M, Langford K. The underreporting of lightning injuries and deaths in Colorado. Bull Am Meteor Soc. 1993;74:2171–8. 10.1175/1520-0477(1993)074<2171:tuolia>2.0.co;2.

[7] Browne BJ, Gaasch WR. Electrical injuries and lightning. Emerg Med Clin North Am. 1992;10:211–29. 1559466

[8] Ritenour AE, Morton MJ, McManus JG, Barillo DJ, Cancio LC. Lightning injury: a review. Burns. 2008;34:585–94. 10.1016/j.burns.2007.11.006 18395987

[9] Centers for Disease Control and Prevention (CDC). Lightning-associated deaths–United States, 1980–1995. MMWR Morb Mortal Wkly Rep. 1998;47:391–4. 9614008

[10] Berko J, Ingram DD, Saha S, Parker JD. Deaths attributed to heat, cold, and other weather events in the United States, 2006–2010. Natl Health Stat Rep. 2014;76:1-15. 25073563

[11] McIntyre WF, Simpson CS, Redfearn DP, Abdollah H, Baranchuk A. The lightning heart: a case report and brief review of the cardiovascular complications of lightning injury. Indian Pacing Electrophysiol J. 2010;10:429–34. PMC2933371 20930961

[12] Blumenthal R. The explosive effects of lightning: what are the risks? Acad Forensic Pathol. 2016;6:89–95. 10.23907/2016.008 PMC6474511 31239875

[13] Cooper MA. Lightning injuries: prognostic signs for death. Ann Emerg Med. 1980;9:134–8. 10.1016/s0196-0644(80)80268-x 7362103

[14] Pereda AE. Electrical synapses and their functional interactions with chemical synapses. Nat Rev Neurosci. 2014;15(4):250–63. 10.1038/nrn3708 PMC4091911 24619342

[15] Cooper MA, Holle RL, Andrews CJ. Distribution of lightning injury mechanisms. In: 30th International Conference on Lightning Protection (ICLP), 13-17 Sep 2010, Cagliari, Italy. 10.1109/iclp.2010.7845948.

[16] Cooper MA. A fifth mechanism of lightning injury. Acad Emerg Med. 2002;9:172–4. 10.1111/j.1553-2712.2002.tb00237.x. 11825846

[17] Blumenthal R. Lightning and the forensic pathologist. Acad Forensic Pathol. 2018;8:98–111. 10.23907/2018.007 PMC6474460 31240028

[18] Fontanarosa PB. Electrical shock and lightning strike. Ann Emerg Med. 1993;22:378–87. 10.1016/s0196-0644(05)80468-8. 8434837

[19] Lichtenberg R, Dries D, Ward K, Marshall W, Scanlon P. Cardiovascular effects of lightning strikes. J Am Coll Cardiol. 1993;21:531–6. 10.1016/0735-1097(93)90699-2 8426021

[20] Cherington M, Wachtel H, Yarnell PR. Could lightning injury be magnetically induced? Lancet. 1998;351:1788. 10.1016/S0140-6736(05)78750-X 9635960

[21] Andrews CJ, Reisner AD. Neurological and neuropsychological consequences of electrical and lightning shock: review and theories of causation. Neural Regen Res. 2017;12:677–86. 10.4103/1673-5374.206636 PMC5461597 28616016

[22] Cherington M, Yarnell PR, London SF. Neurologic complications of lightning injuries. West J Med. 1995;162:413–7. PMC1022790 7785254

[23] Huan-Jui Y, Chih-Yang L, Huei-Yu L, Po-Chih C. Acute ischemic stroke in low-voltage electrical injury: a case report. Surg Neurol Int. 2010;1:83. 10.4103/2152-7806.74093 PMC3011102 21206535

[24] Eriksson A, Ornehult L. Death by lightning. Am J Forensic Med Pathol. 1988;9:295–300. 10.1097/00000433-198812000-00005 3239547

[25] McAleese KE, Alafuzoff I, Charidimou A, *et al*. Post-mortem assessment in vascular dementia: advances and aspirations. BMC Med. 2016;14:129. 10.1186/s12916-016-0676-5 PMC5011905 27600683

[26] Jain S, Bandi V. Electrical and lightning injuries. Crit Care Clin. 1999;15:319–31. 10.1016/s0749-0704(05)70057-9 10331131

[27] Gluncić I, Roje Z, Gluncić V, Poljak K. Ear injuries caused by lightning: report of 18 cases. J Laryngol Otol. 2001;115:4–8. 10.1258/0022215011906858 11233621

[28] Cherington M, Yarnell P, Hallmark D. MRI in lightning encephalopathy. Neurology. 1993;43:1437–8. 10.1212/wnl.43.7.1437 8327155

